# Editorial: Recurrent pregnancy loss and endocrine dysfunction

**DOI:** 10.3389/fendo.2024.1453336

**Published:** 2024-07-09

**Authors:** Hong Zhang, Lianghui Diao

**Affiliations:** ^1^ Department of Obstetrics and Gynecology, the Second Affiliated Hospital of Soochow University, Suzhou, China; ^2^ Shenzhen Key Laboratory of Reproductive Immunology for Peri-implantation, Shenzhen Zhongshan Institute for Reproductive Medicine and Genetics, Shenzhen Zhongshan Obstetrics & Gynecology Hospital, Shenzhen, China

**Keywords:** thyroid dysfunction, luteal phase deficiency, diabetes mellitus, insulin resistance, recurrent pregnancy loss, IVF/ICSI

Recurrent pregnancy loss (RPL) is a distressing condition affecting a significant number of women globally, with complex etiologies that often remain elusive (1). It is estimated that approximately 8% to 12% of all cases of RPL are caused by endocrine diseases, which include thyroid dysfunction, luteal phase deficiency, hyperprolactinemia, diabetes mellitus, insulin resistance, polycystic ovarian syndrome (PCOS), and so on (2). The Research Topic “Recurrent Pregnancy Loss and Endocrine Dysfunction”, aims to deepen our understanding of how endocrine issues contribute to RPL and to identify potential therapeutic targets.

The manuscripts accepted for this Research Topic present a diverse range of studies, each providing valuable insights into different aspects of endocrine-related RPL.

The cross-sectional study by Zhang et al. highlights the high prevalence of endocrine dysfunctions, such as thyroid dysfunction, hyperprolactinemia, obesity, PCOS, and glucose abnormalities in women with RPL. They emphasize the importance of comprehensive endocrine evaluations of endocrine dysfunction in recurrent pregnancy loss, proposing that obesity may be a key endocrine factor among patients with two or more pregnancy losses, and suggesting screening of patients for endocrine-related etiology after two miscarriages.

Several studies focus on specific endocrine disorders and their impact on RPL.

Huang et al. investigates the effect of thyroid-stimulating hormone (TSH) levels post-controlled ovarian hyperstimulation on IVF/ICSI outcomes. It finds that while TSH levels do not significantly affect pregnancy rates, lower TSH levels are associated with higher preterm delivery rates.

In terms of treatment strategies, sequential embryo transfer could improve the clinical outcomes of patients with recurrent implantation failure (Gao et al.). Fang et al. found that the number of previous embryo implantation failures is an independent factor affecting implantation rate, clinical pregnancy rate, spontaneous abortion rate and live birth rate of patients underwent IVF/ICSI- ET.

Adenomyosis can induce heavy menstrual bleeding, chronic pelvic pain, infertility and RPL. Ge et al. explore the effects of ovarian stimulation protocols on assisted reproductive technology outcomes in women with adenomyosis, they recommended that an ultra-long or long protocol might be beneficial for fresh embryo transfer.

The link between lupus and RPL is explored in another study, which finds that women with lupus have a higher risk of pregnancy loss due to the autoimmune nature of the disease, adding to the complexity of managing RPL in these patients (Valeff et al.).

Ren et al. found that exposure to environmental pollutants is also a risk factor for RPL. Insulin resistance, vitamin D insufficiency, and abnormal thyroid and sex hormone concentrations might contribute to heavy metal-related abortion.

Low follistatin levels have also been identified as a potential risk factor for RPL. This study indicates that follistatin may play a protective role in maintaining pregnancy, highlighting another endocrine factor that could be crucial in managing RPL (Gong et al.).

Li et al. provide a metabolic perspective by analyzing follicular fluid in patients with diminished ovarian reserve (DOR), showing unique metabolic characteristics that could be associated with RPL.

## Conclusion

In conclusion, these studies collectively enhance our understanding of the multifaceted relationship between endocrine dysfunction and RPL. They underscore the importance of comprehensive and individualized endocrine evaluations in women with RPL to improve clinical outcomes. The knowledge gained from this compilation of studies aims to reduce the burden of RPL on affected women and their families, ultimately leading to better clinical practices and improved reproductive health outcomes.

We express our gratitude to all contributors for their invaluable research and to the reviewers for their critical evaluations. We hope this Research Topic will stimulate further research and enhance clinical practices in managing endocrine-related recurrent pregnancy loss.

## Author contributions

HZ: Writing – review & editing. LD: Writing – original draft.

## References

[1] Quenby S Gallos ID Dhillon-Smith RK Podesek M Stephenson MD Fisher J. Miscarriage matters: the epidemiological, physical, psychological, and economic costs of early pregnancy loss. Lancet. (2021) 397:1658–67. doi: 10.1016/S0140-6736(21)00682-6 33915094

[2] Pluchino N Drakopoulos P Wenger JM Petignat P Streuli I Genazzani AR. Hormonal causes of recurrent pregnancy loss (RPL). Hormones (Athens). (2014) 13:314–22. doi: 10.14310/horm.2002 25079455

